# Dataset of computer science course queries from students: Categorized and scored according to Bloom's taxonomy

**DOI:** 10.1016/j.dib.2024.110109

**Published:** 2024-02-01

**Authors:** Khandoker Ashik Uz Zaman, Ashraful Islam, Yusuf Mahbubul Islam, Md Abu Sayed

**Affiliations:** a Department of Computer Science and Engineering, Independent University Bangladesh, Dhaka 1229, Bangladesh; b Center for Computational & Data Sciences, Independent University Bangladesh, Dhaka 1229, Bangladesh

**Keywords:** Education, Academic learning, Questioning, Computer science, Artificial intelligence, Machine learning

## Abstract

“Why don't students learn?” is a common question that educators try to address. To encourage students to become more engaged in the learning process, we believe in fostering their natural curiosity by encouraging them to ask high-level questions. To support this approach, we have compiled a dataset of questions that we hope will aid in the training of artificial intelligence (AI) models and ultimately improve the learning experience for students. To develop our dataset, we collected anonymous student questioning data in the Summer 2023 semester, utilizing our online application named “Palta Question”, resulting in a dataset of 8,811 unique questions. The dataset consists of students’ inquiries which underwent basic question validation using a sophisticated keyword-based approach, manual categorization by topic and course content, as well as complexity assessment using Bloom's taxonomy keywords which have also been included in the dataset. To ensure question uniqueness, we implemented the Levenshtein distance algorithm to exclude questions with a high similarity rate. This dataset provides targeted insights into student inquiry patterns and knowledge gaps within the domain of 'Introduction to Computers and Research' and 'Data Structure' courses, originating from the students at Independent University, Bangladesh (IUB). While its scope is confined to a specific student group and academic context, limiting broader applicability, it remains valuable for detailed studies in these subjects and serves as a useful foundation for AI-based educational research tools. To demonstrate the effectiveness of the dataset, we also tested it to train the AI to perform basic tasks like sorting questions according to their courses and topics. However, we envision researchers utilizing it to enhance education and aid in students' learning.

Specifications Table

**Table:** 

Subject	Computer Science Applications, Education
Specific subject area	Categorized and scored questions from undergraduate students enrolled in computer science courses.
Data format	Raw, Analyzed, Processed, Filtered
Type of data	Comma Separated Value (.CSV)
Data collection	The data collection for this dataset was conducted using an online application called “Palta Question”. The dataset contains manually extracted 8811 unique questions from a total of 9250. The questions were categorized based on topic and course after processing them. Finally, these questions were scored by an algorithm using Bloom's Taxonomy keywords. The dataset was aimed to gather data from computer science students enrolled in two different courses: one technical course on “Data Structures”, and one less technical course on “Introduction to Computers and Research”.
Data source location	The data was collected from undergraduate students at Independent University, Bangladesh (IUB), Dhaka, Bangladesh studying in computer science courses.
Data accessibility	The data is published in Mendeley Data. Repository name: Dataset of Computer Science Course Queries from Students: Categorized and Scored According to Bloom's Taxonomy Data identification number: 10.17632/w5zt9n6vsc.4 Direct URL to data: https://data.mendeley.com/datasets/w5zt9n6vsc/3

## 1 Value of the Data

• Getting students to ask questions is a big challenge and getting them to ask high-level questions is a greater challenge. By analyzing the dataset, researchers can gain insights into effective strategies for nurturing students' curiosity and promoting active inquiry-based learning, addressing the challenge of stimulating high-level questioning among students.
• The dataset's well-structured nature, including question rankings and categorization, makes it a valuable resource for training artificial intelligence (AI) models. Researchers and developers working on machine learning (ML) and AI applications for education can leverage it to enhance the capabilities of AI-driven educational tools.
• The dataset provides formatted and processed questions, facilitating in-depth analysis. With questions ranked on a numerical scale (from 0 to 150) and categorized by relevance, researchers can easily extract meaningful insights and use the data for various research applications such as question quality assessment and learning pattern analysis.
• Beyond its immediate application, this dataset empowers researchers to gain deeper insights into student learning behavior, question patterns, and knowledge gaps. It serves as a foundation for improving educational strategies and enhancing the overall learning experience for students.
• By gathering Bloom's Taxonomy keywords, which are not readily available from a single source, this dataset addresses a critical gap in educational resources. It highlights the complexity of keywords appearing at multiple cognitive levels, a challenge in query evaluation. This dataset makes it easier for researchers to evaluate academic queries effectively, particularly in the field of computer science.

## 2 Background

The central challenge in education today is to cultivate students' curiosity and actively engage them in the learning process [1]. Encouraging students to ask questions is a crucial aspect of this challenge [2]. Research shows that students' inquiries are not only a sign of their curiosity but also a powerful tool for grasping complex concepts and honing critical thinking skills [3]. However, educators often struggle as students’ questions deviate from the subject matter [4]. To address this challenge, we created a dataset of questions that can help in training AI and ML models to categorize questions effectively, distinguishing between relevant and irrelevant inquiries and helping improve education and learning further by analyzing these questions.

Our goal behind this dataset is to improve the quality of education through technological innovation. By training AI models to identify and categorize questions accurately, we aim to streamline the learning process and enable educators to focus on delivering targeted and relevant content. Moreover, this dataset may have a potentially wider range of research applications, from content gap analysis and exam question generation to AI-driven educational tools and predictive models for student performance.

### 2.1 Data description

The data was collected from 126 students at Independent University, Bangladesh (IUB). Collected data are fully anonymized. The dataset is publicly available on Mendeley [5]. The dataset consists of four Comma Separated Values (CSV) files as follows:

The “Data_Structure.csv” file, the “Introduction_to_Computers_and_Research.csv” file, and the “Irrelevant_Questions.csv” file consist of the questions asked by students in the classroom using an online application. The topics associated with the questions are stored in the “Questions” column and the “Score” column contains the points associated with the question. The “Irrelevant_Questions.csv” file contains the questions that were asked in the “Data Structure” and “Introduction to Computers and Research” courses but were not related to the course topics. This file does not contain any topics associated with the questions as the questions are very diverse and contain a lot of unrelated topics which the students asked questions on as their minds diverted away from the class.

All the questions in the dataset are scored by an algorithm employing Bloom's Taxonomy [6] keywords. The set of keywords has been curated and compiled from multiple sources. In cases where the same keyword appeared at different levels in the taxonomy, we prioritized the level where it was most commonly associated. The “Blooms_Taxonomy.csv” file contains a set of keywords that are organized based on the different levels of the taxonomy. Each row in the file contains different keywords (verbs) along with all their forms. For instance, the word “allow” has all its forms - “allowed,” “allows,” and “allowing” included in the dataset. Each of the columns represents the different levels of the taxonomy.

**Fig. 1:**
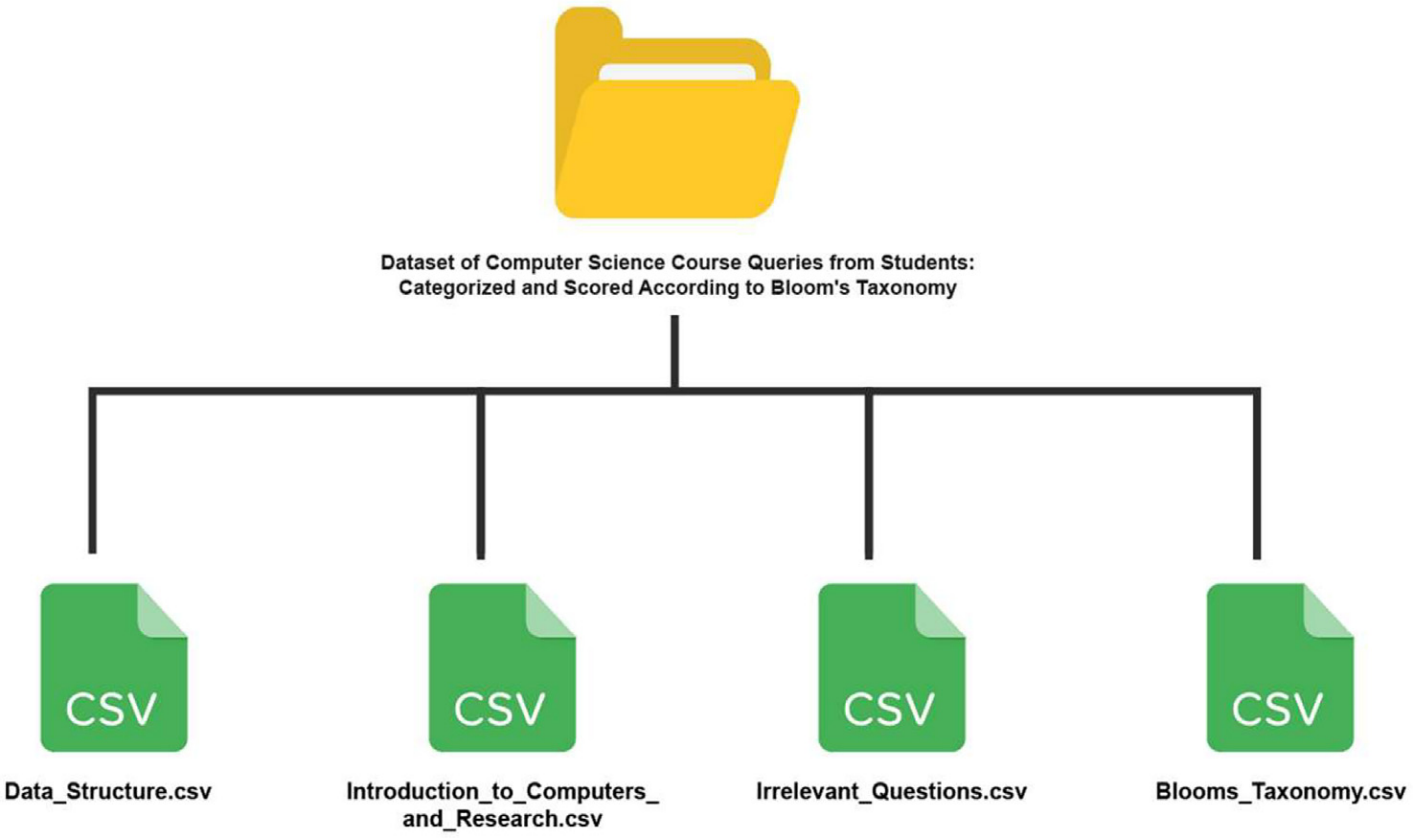
The file structure of the dataset containing four CSV files.

Fig. 1 illustrates the file structure of the dataset. Table 1 presents the description of each files available in the dataset. Descriptions of contents recorded in CSV files are summarized in Table 2, Table 3, Table 4.

**Table 1:** File description of the dataset: “Dataset of computer science course queries from students: categorized and scored according to bloom's taxonomy”.

File Name	File Description	No. of Columns	File Size
Data_Structure.csv	This file contains the scored and categorized questions from the “Data Structure” course.	3	152 KB
Introduction_to_Computers_and_Research.csv	This file contains the scored and categorized questions from the “Introduction to Computers and Research” course.	3	145 KB
Irrelevant_Questions.csv	This file contains irrelevant questions that do not belong to the courses above but were asked by the students from those courses.	2	561 KB
Blooms_Taxonomy.csv	This file contains the Bloom's Taxonomy keywords that were used to evaluate the questions.	6	14.7 KB

**Table 2:** Content description of “Data_Structure.csv” and “Introduction_to_Computers_and_Research.csv” files.

Column No.	Column Name	Data Type	Description
1	Topic	String	The topic of the question
2	Score	Integer (0 to 100)	Level/Rank of the question
3	Questions	String	Pre-processed question

**Table 3:** Content description of “Irrelevant_Questions.csv.” file.

Column No.	Column Name	Data Type	Description
1	Score	Integer (0 to 100)	Level/Rank of the question
2	Questions	String	Pre-processed question

**Table 4:** Content description of “Blooms_Taxonomy.csv.” file.

Column No.	Column Name	Data Type	Description
1	Remember	String	Level 1 Keywords (Lowest Level)
2	Understand	String	Level 2 Keywords
3	Apply	String	Level 3 Keywords
4	Analyze	String	Level 4 Keywords
5	Evaluate	String	Level 5 Keywords
6	Create	String	Level 6 Keywords (Highest Level)

## 3 Experimental Design, Materials and Methods

### 3.1 Study design

**Fig. 2:**
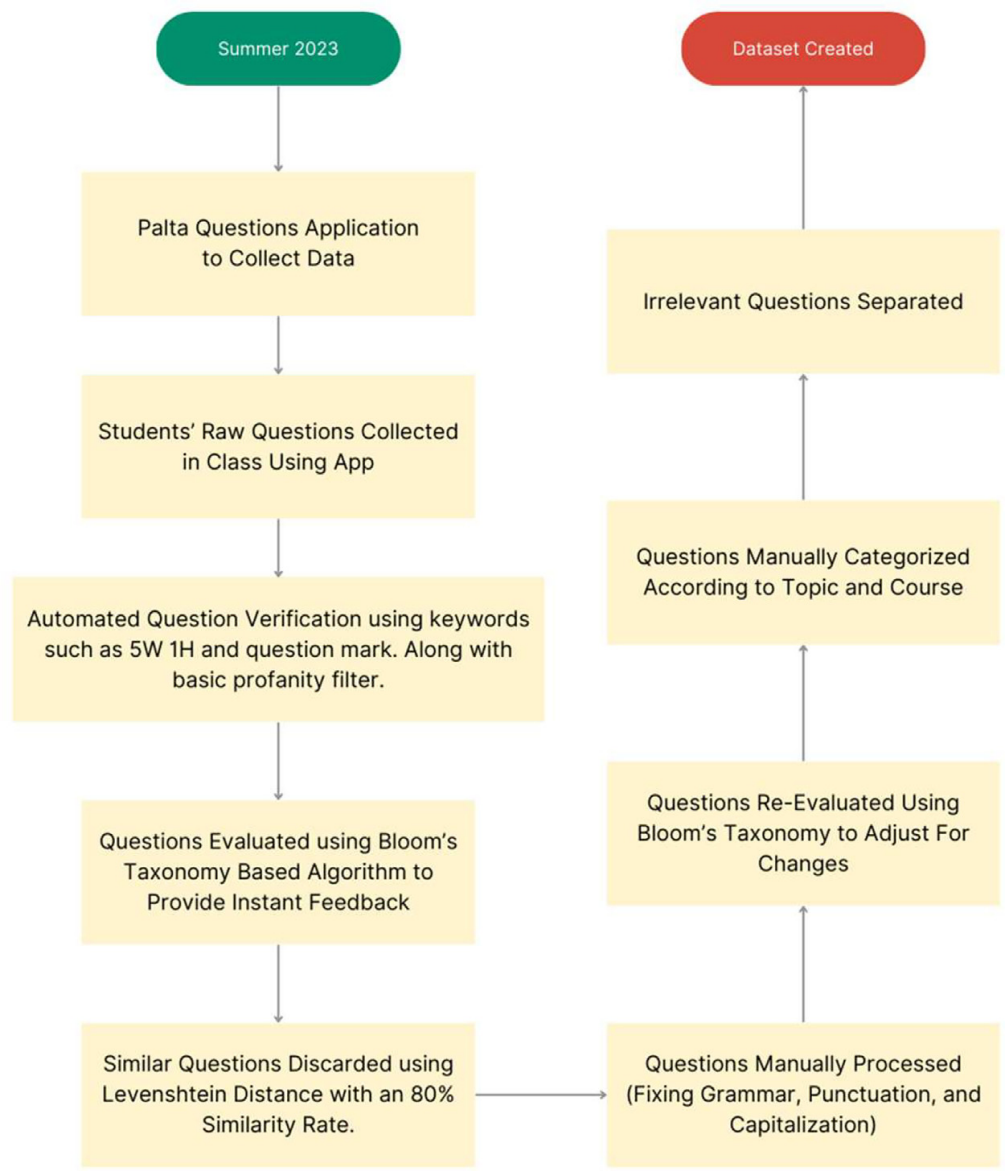
Dataset creation process.

The research was conducted on computer science students who were enrolled in either one of the two computer science courses: one technical course on “Data Structures”, and one less technical course on “Introduction to Computers and Research” to get a more diverse dataset of questions. Their questions have been recorded throughout the Summer 2023 Semester (June 2023 – August 2023). The students were allowed to ask questions both before and after the class once the topic had been introduced. This allowed them to clear any doubts they had before the lecture, focus on the topic during the class activities and lectures, and later use that knowledge to ask further questions. This allowed students to progress from basic to advanced questioning, with their skill advancement being more visible. Fig. 2 illustrates the whole dataset creation process sequentially.

### 3.2 Participants

A total of 126 students (male=85 and female=41) participated in this research. The consent of participants was taken, and the data was collected anonymously. The group consisted of individuals from varied backgrounds, including both newcomers and senior students due to the open credit system at IUB.

### 3.3 Data collection

**Fig. 3:**
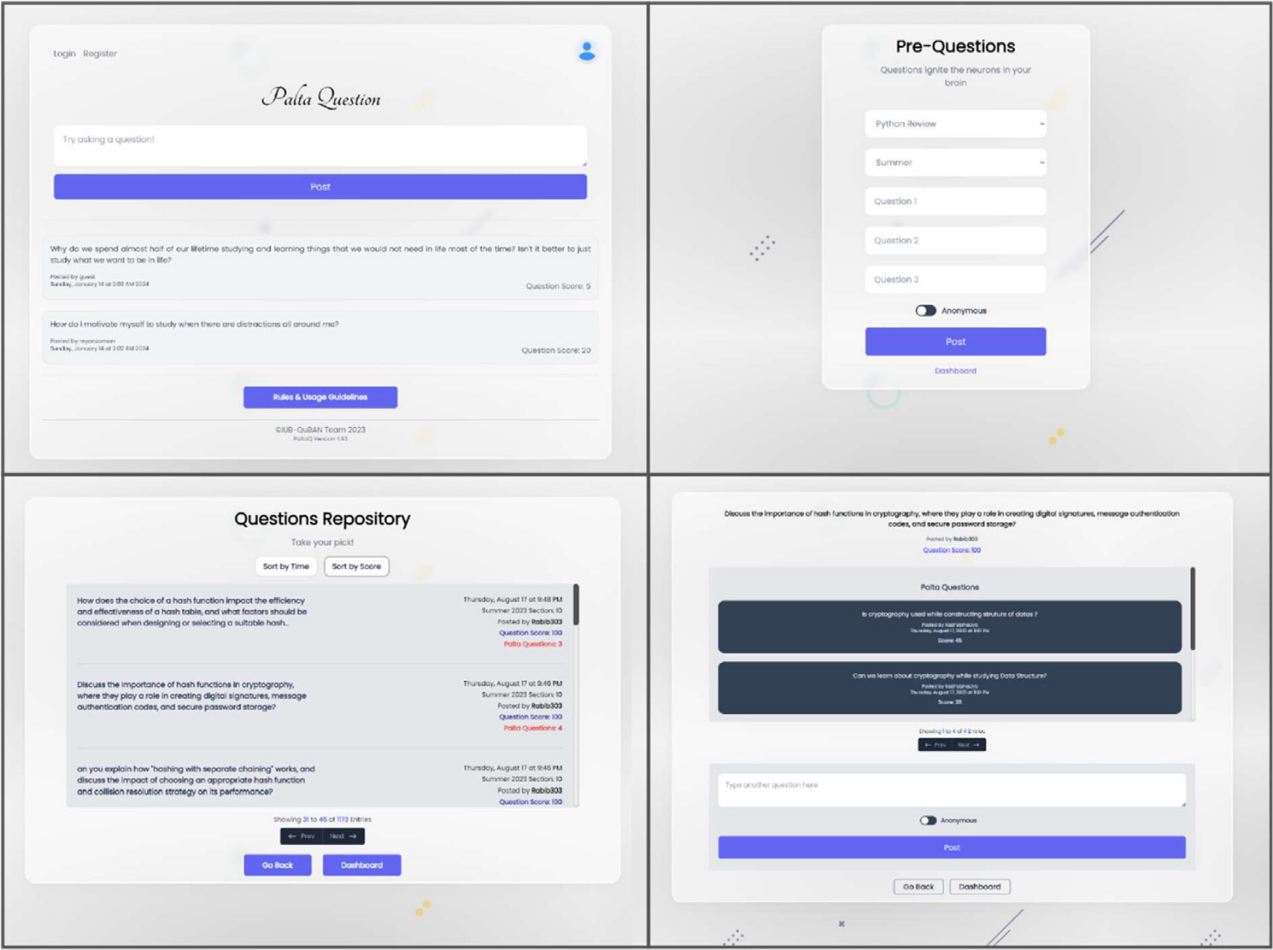
The interfaces of the “Palta Question” application which was used to collect the data from students where they either anonymously questions as guest users or used an alias.

The data collection procedure included collecting data anonymously using our application called “Palta Question” which we modeled for this research. The application is a web-based application that students use in the first 10 min of the class and after the class at their home. The application had an interface that enabled students to select the topic they wished to ask questions about. However, it was often observed that students did not ask questions related to the topic they had chosen. Therefore, it became necessary to process the data before entering it into the dataset. Fig. 3 shows the user interfaces of the “Palta Question” application.

### 3.4 Data processing

All the raw questions were first analyzed, and duplicate questions were removed by eliminating all questions using Levenshtein Distance [7] with a similarity rate of 80 %. From this, we found 8811 unique filtered questions that underwent manual processing by fixing grammar, punctuation, and capitalization. The processed questions were re-evaluated using the keyword-based algorithm based on Bloom's Taxonomy to adjust for any change that happened during the processing stage. These questions were manually categorized according to their topics and courses. The irrelevant questions that were asked by students from the selected courses but not related to the topic were manually separated from the questions belonging to the two selected courses.

### 3.5 Data evaluation and scoring

**Fig. 4:**
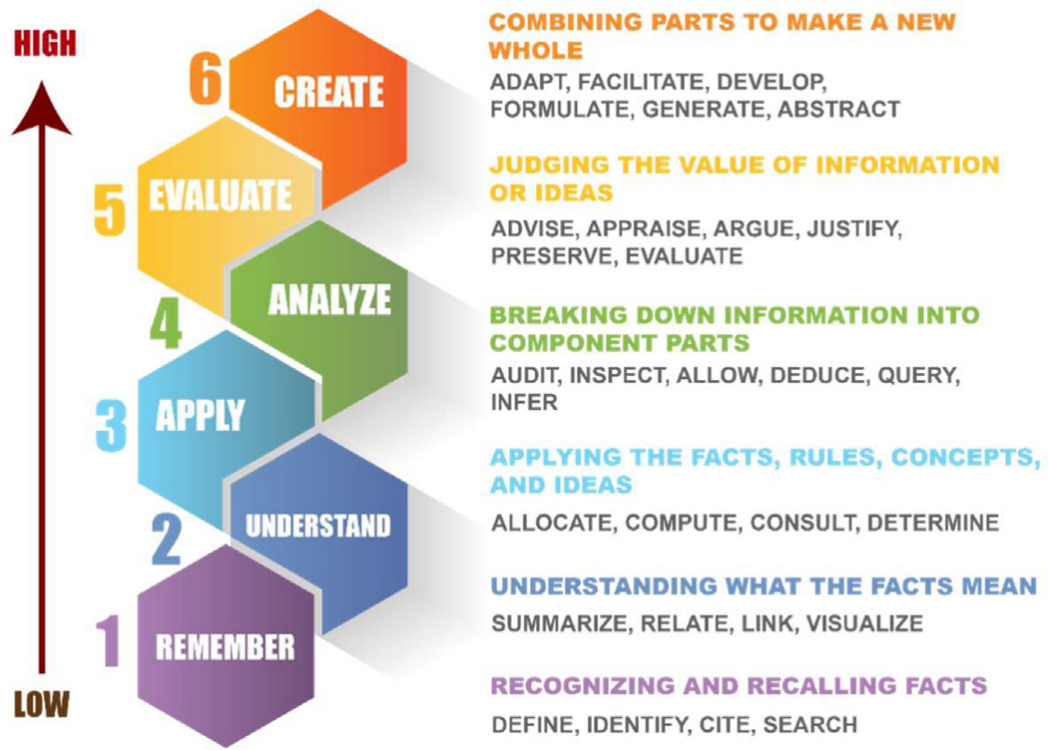
Levels of Bloom's taxonomy and some associated keywords.

Instead of stemming and lemmatization, which can be quite inaccurate and computationally expensive, we have manually listed down all the different forms of the verbs in Bloom's Taxonomy. Fig. 4 presents the levels of Bloom's taxonomy and some associated keywords. After matching the keywords on Bloom's taxonomy levels with the individual words in the questions, we have assigned a score to each question based on the level.

**Fig. 5:**
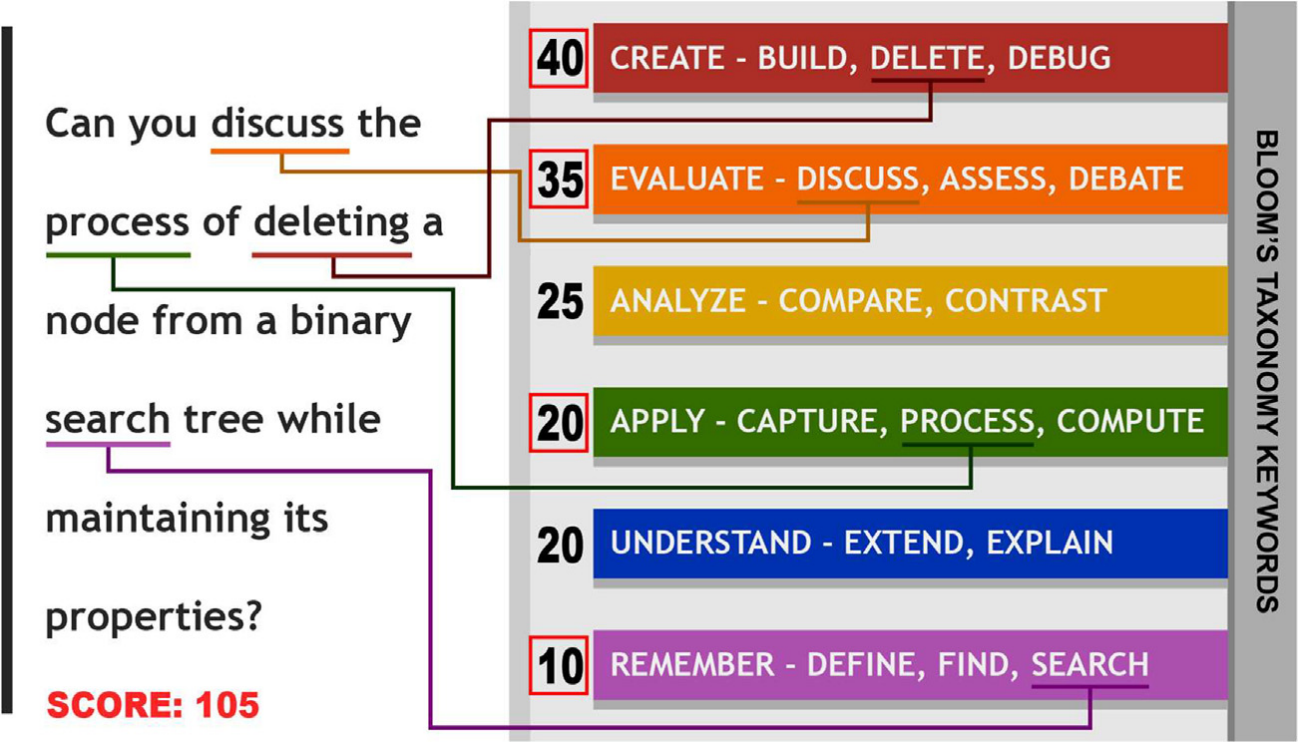
Question evaluation system.

The higher levels of Bloom's taxonomy indicate a higher level of the question so, we have associated greater points with the upper levels of the taxonomy. Formulating a question using keywords from the top three levels gives a score of 100 points which indicates that questions above 100 points can be considered high-level questions. A single question can score a maximum of 150 points which is the highest level of a question according to this algorithm. A demonstration of the question evaluation system is shown in Fig. 5.

The scoring system was calculated according to Table 5 from Level 1 (Lowest level) to Level 6 (Highest level). The points have been carefully planned and distributed to the different levels of the taxonomy to align with the theoretical aspect of Bloom's Taxonomy.

**Table 5:** Levels of Bloom's Taxonomy and associated scores.

Level 1	Level 2	Level 3	Level 4	Level 5	Level 6
Knowledge-Based Questions	Comprehensive Questions	Application-Based Questions	Analytical Questions	Evaluative Questions	Synthetic Questions
10 points	20 points	20 points	25 points	35 points	40 points

The Python code for the question evaluation system and data processing is publicly available to use. The code is available on GitHub [8].

## Limitations

This dataset offers valuable insights for educational research and AI applications, but its creation and usage come with challenges and limitations. Limited by the number of participating students, the dataset mainly represents the questioning behavior of a specific group at IUB. Thus, generalizing findings to a broader population requires caution. Its focus on only “Introduction to Computers and Research” and “Data Structure” courses also limits its applicability across other academic areas.

A significant portion of student inquiries were off-topic, and some questions might have been copied from online sources by the participants, affecting originality and relevance. Additionally, in Bloom's Taxonomy, the same keyword can appear at different levels, and in this dataset, keywords are categorized based on their most common usage in computer science, not by specific context. The current keyword-based detection could be enhanced with context-based detection using AI for more accurate interpretations.

Students’ challenges in articulating their questions clearly also posed a limitation, leading to vague or poorly structured inquiries. The dataset required extensive manual pre-processing, including question validation and categorization. Future improvements could automate these processes, enhancing scalability and minimizing human bias in question selection.

## Ethics Statement

The authors have complied with the ethical requirements for publication in the Data in Brief journal. This study involved human participants for obtaining feedback (questions) but did not involve any experiments with human subjects, animals, or any data collected from social media platforms. This study was conducted ethically adhering to the guidelines within a classroom environment and was exempted from ethical review. However, the participants consented to the use of the application that was used to collect the data. Participants in the research process were given the freedom to quit at any time.

## CRediT authorship contribution statement

**Khandoker Ashik Uz Zaman:** Conceptualization, Methodology, Writing – original draft, Formal analysis, Data curation. **Ashraful Islam:** Conceptualization, Supervision, Writing – review & editing. **Yusuf Mahbubul Islam:** Conceptualization, Methodology, Supervision. **Md Abu Sayed:** Conceptualization, Methodology, Validation, Supervision.

## Data Availability

Dataset of Computer Science Course Queries from Students: Categorized and Scored According to Bloom's Taxonomy (Original data) (Mendeley Data)
